# Zebularine suppresses TGF-beta-induced lens epithelial cell–myofibroblast transdifferentiation by inhibiting MeCP2

**Published:** 2011-10-19

**Authors:** Peng Zhou, Yi Lu, Xing-Huai Sun

**Affiliations:** Department of Ophthalmology, Eye and ENT Hospital of Fudan University, Shanghai, China

## Abstract

**Purpose:**

Posterior capsular opacification (PCO) is a common long-term complication of modern cataract surgery. Remnant lens epithelial cells (LECs) undergo a myofibroblast transdifferentiation that is thought to be the initial step of PCO pathogenesis. The purpose of this study is to determine the effects of zebularine on transforming growth factor-β (TGFβ)-induced, LEC-myofibroblast transdifferentiation.

**Methods:**

The expression levels of methyl CpG binding protein 2 (MeCP2) and α-smooth muscle actin (α-SMA) in human PCO membranes were evaluated by confocal microscopy. The role that MeCP2 played in TGFβ2-induced α-SMA expression was analyzed by western blotting both before and after *MeCP2* knockdown with *MeCP2*-specific siRNA. The effect of zebularine on *MeCP2* expression was analyzed over time using a variety of dosages. The effect of zebularine on TGFβ2-induced α-SMA expression was determined by western blot analysis.

**Results:**

MeCP2 and α-SMA co-localized in human PCO membranes. When MeCP2 was depleted, TGFβ2 could not induce α-SMA expression. Zebularine decreased *MeCP2* expression in lens epithelial cells in a time- and dose-dependent pattern and reversed TGFβ2-induced α-SMA expression.

**Conclusions:**

MeCP2 plays an important role in TGFβ2-induced α-SMA expression in lens epithelial cells. Zebularine could reverse the TGFβ2-induced α-SMA expression by inhibiting *MeCP2* expression. Therefore, zebularine could potentially prevent PCO formation.

## Introduction

Posterior capsular opacification (PCO), also known as secondary cataract, is a common long-term complication of modern cataract surgery [1,2]. Decreased visual acuity induced by PCO is reported to occur in 20% to 40% of patients two to five years after surgery [2,3]. Presently, the only effective treatment of PCO is Nd:YAG laser capsulotomy. However, complications include retinal detachment, damage to the intraocular lens, and cystoid macular edema [4,5]. Therefore, a better understanding of the pathogenic mechanism of PCO and effective treatments to prevent PCO are highly desirable.

PCO is caused mainly by remnant lens epithelial cells (LECs), which undergo an epithelial cell-myofibroblast transdifferentiation (EMT), followed by enhanced proliferation, migration, and collagen deposition. During the process of epithelial cell-myofibroblast transdifferentiation, an epithelial cell undergoes phenotypic changes to resemble a mesenchymal cell and expresses alpha smooth muscle actin (α-SMA) as a molecular marker. Although inflammatory, fibrogenic growth factors and cytokines produced by injured tissues help to orchestrate the process of EMT, transforming growth factor β (TGFβ) is believed to play a central role in the process [6-9]. Recently, it has been reported that DNA methylation is an important mechanism of the *α-SMA* gene expression during myofibroblast differentiation, suggesting that the epithelial cell-myofibroblast transdifferentiation is also regulated by epigenetic factors [10].

Epigenetic regulation of gene expression commonly occurs at two primary levels: DNA methylation and histone modifications. Methyl CpG binding protein 2 (MeCP2), a key member of the methyl-DNA binding protein family of proteins, has been suggested as essential for myofibroblast differentiation [11]. While MeCP2 can bind to unmethylated DNA, it preferentially binds to methylated DNA at 5′-CpG residues [11]. MeCP2 was originally considered a transcriptional repressor but was later found also to have a significant role as a transcriptional activator. In addition, MeCP2 functions in the regulation of chromatin architecture and RNA splicing [12].

Zebularine (1-(b-D-ribofuranosyl)-1,2-dihydropyrimidin-2-one) has been established as a novel inhibitor of DNA methyltransferase (DNMT) [13]. In contrast to other DNMT inhibitors, it is quite stable [14,15] and has low toxicity [16-18]. Preclinical studies using zebularine have shown favorable toxicity and stability profiles, making it an attractive candidate for epigenetic treatment of PCO [17].

In the present study, we investigated the role of MeCP2 in LEC epithelial cell-myofibroblast transdifferentiation. The effects of zebularine on *MeCP2* expression and TGFβ2-induced LEC epithelial-mesenchymal transitions were analyzed. The purpose of this study was to investigate whether zebularine can be used to prevent PCO.

## Methods

The institutional review board (IRB) of the Fudan University Eye and ENT Hospital (Shanghai, P.R. China) approved our use of PCO membrane from donated eyes and cultured human lens epithelial cells (LECs). All procedures conformed to the Declaration of Helsinki for research involving human subjects. Zebularine was a kind gift from Dr. Victor E. Marquez (Laboratory of Medicinal Chemistry, National Cancer Institute, Frederick, MD).

### Immunofluorescent staining

Three posterior capsular membranes were obtained from donated eyes with PCO one year after cataract surgery. Immunofluorescent staining was performed as previously described [19]. In brief, tissues were snap-frozen and sectioned at 6 μm using a cryostat. Thawed tissue sections were air-dried and rehydrated with PBS (pH 7.4). The slides were fixed with 3.7% paraformaldehyde for 30 min and then rinsed in PBS twice for 10 min. After blocking with 5% normal goat serum for 60 min, the sections were incubated with primary rabbit anti-human MeCP2 (Abcam, Cambridge, MA) at 4 °C overnight. Binding of the primary antibody was visualized with FITC-conjugated anti-rabbit secondary antibody (Vector Laboratories, Burlingame, CA) for 30 min. For double staining, sections were washed and then incubated with mouse monoclonal anti-α-SMA (Abcam) for 1 h at room temperature. After washing, sections were incubated with rhodamine-conjugated anti-mouse secondary antibody (Vector Laboratories). Sections were mounted in a mounting medium (Vectashield; Vector Laboratories), and the sections were examined with a confocal microscope (LSM510; Zeiss, Thornwood, NY) using the Zeiss image acquisition and analysis software (LSM version 3.2). FITC staining was captured using a 488-nm argon laser for excitation and a 505- 530-nm band-pass emission filter. Rhodamine staining was captured using a 543-nm HeNe laser for excitation and a 560-nm long-pass emission. Negative controls included omitting the primary antibody and the use of IgG in place of the primary antibody (at the same concentration).

### Cell culture

HLE B-3 cells were obtained from ATCC (Rockville, MD) and cultured in Eagle’s minimum essential medium (GIBCO BRL, Grand Island, NY) with 20% fetal bovine serum, 100 units/ml penicillin, and 100 mg/ml streptomycin at 37 °C in a humidified 5% CO2 atmosphere.

### siRNA transfection

siRNA for *MeCP2* (SASI_Hs01_00116141) and non-silencing control scrambled RNA (Mission-SIC-001-s) were purchased from Sigma–Aldrich (St. Louis, MO). siRNA was transfected using the Hiperfect Transfection Reagent (Qiagen, Valencia, CA) according to the manufacturer’s instructions. A total of 5×10^5^ cells in 2 ml of medium were seeded in 6-well plates. siRNA (final concentration 10 nM) was then gently introduced into the cells by mixing with the required amount of Hiperfect Transfection Reagent. The assays were performed 72 h post-transfection.

### Western blot assay

Confluent cells grown in 6-well plates in DMEM with 0.4% FBS were lysed, supernatants were then collected, and proteins were resolved on Tris-HCl 10% polyacrylamide gels at 120 V. The proteins were transferred to a PVDF blotting membrane (Millipore, Bedford, MA). The membranes were probed with antibodies for MeCP2 (Abcam), α-SMA (Abcam), and glyceraldehyde 3-phosphate dehydrogenase (GAPDH; Abcam), respectively, all at 1:1,000 dilution. Membranes were washed and incubated with a horseradish peroxidase (HRP)-conjugated secondary antibody (1:3,000; Vector Laboratories) for 30 min at room temperature. Images were developed using an ECL chemiluminescence detection solution (Amersham Pharmacia Biotech, Cleveland, OH).

### Statistics

All experiments were performed at least three times. The data were analyzed using the Student’s *t*-test, and a p<0.05 was accepted as significant.

## Results

### MeCP2 and α-SMA are co-expressed in human PCO membranes

**Figure 1:**
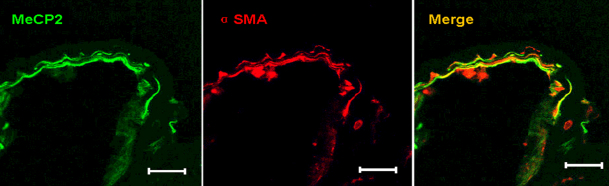
Co-expression of MeCP2 and α-SMA in human PCO membranes. Tissue sections from human PCO membrane were stained by immunofluorescence for MeCP2 and α-SMA and evaluated by confocal microscopy. MeCP2 and α-SMA were expressed in cells within the PCO membrane. Merged images show colocalization of MeCP2 and α-SMA. The scale bar is 50 μm.

We first evaluated the expression of MeCP2 and α-SMA in PCO membranes. Three PCO membranes were evaluated and all showed prominent immunoreactivity for MeCP2. Labeling with anti-α-SMA antibodies revealed numerous α-SMA-positive transdifferentiated LECs within the PCO membrane. MeCP2 and α-SMA staining at least partially overlapped in each membrane, indicating that MeCP2 and α-SMA were co-expressed in the PCO membrane (Figure 1). This result raises the possibility that MeCP2 plays a role in α-SMA expression. Alternatively, MeCP2 and α-SMA expression could occur in the same cells without any causal relationship.

### MeCP2 is required for TGFβ2-induced α-SMA expression

**Figure 2:**
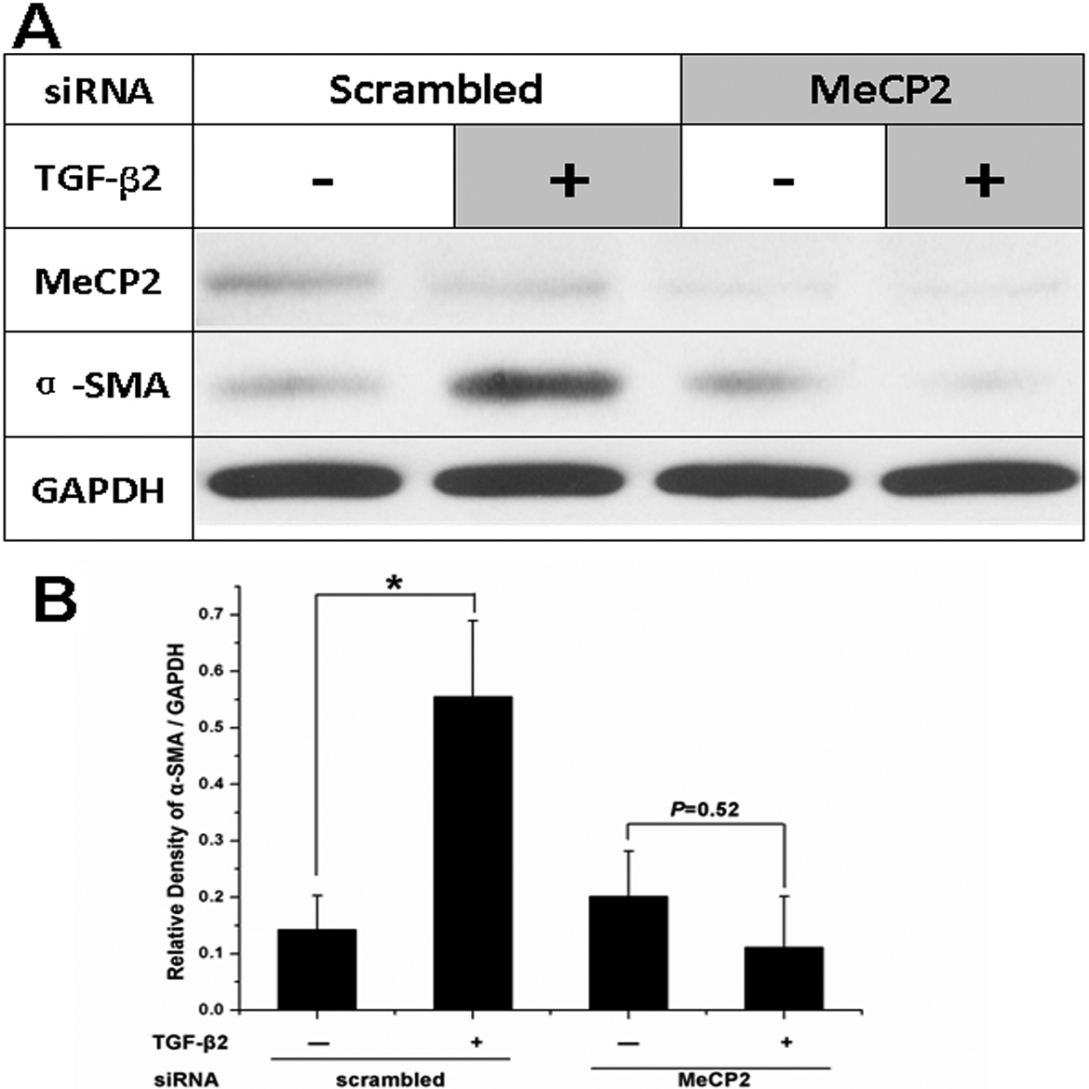
TGFβ2 did not induce α-SMA expression without MeCP2. **A**: TGFβ2 increased α-SMA expression in lens epithelial cells. However, TGFβ2 could not increase the expression of α-SMA after MeCP2 was knocked down using MeCP2 specific siRNA. **B**: Densitometry analysis of three independent western blot analyses shows the quantitative α-SMA. *p<0.05. The error bar indicates mean±SEM.

We next analyzed whether MeCP2 plays a role in TGFβ2-induced α-SMA expression. TGFβ2 increased α-SMA expression in LECs. However, when we knocked down *MeCP2* using *MeCP2*-specific siRNA, TGFβ2 could not increase the expression of α-SMA (Figure 2). Therefore, *MeCP2* plays an important role in TGFβ2-induced α-SMA expression.

### Zebularine decreased MeCP2 expression in lens epithelial cells

**Figure 3:**
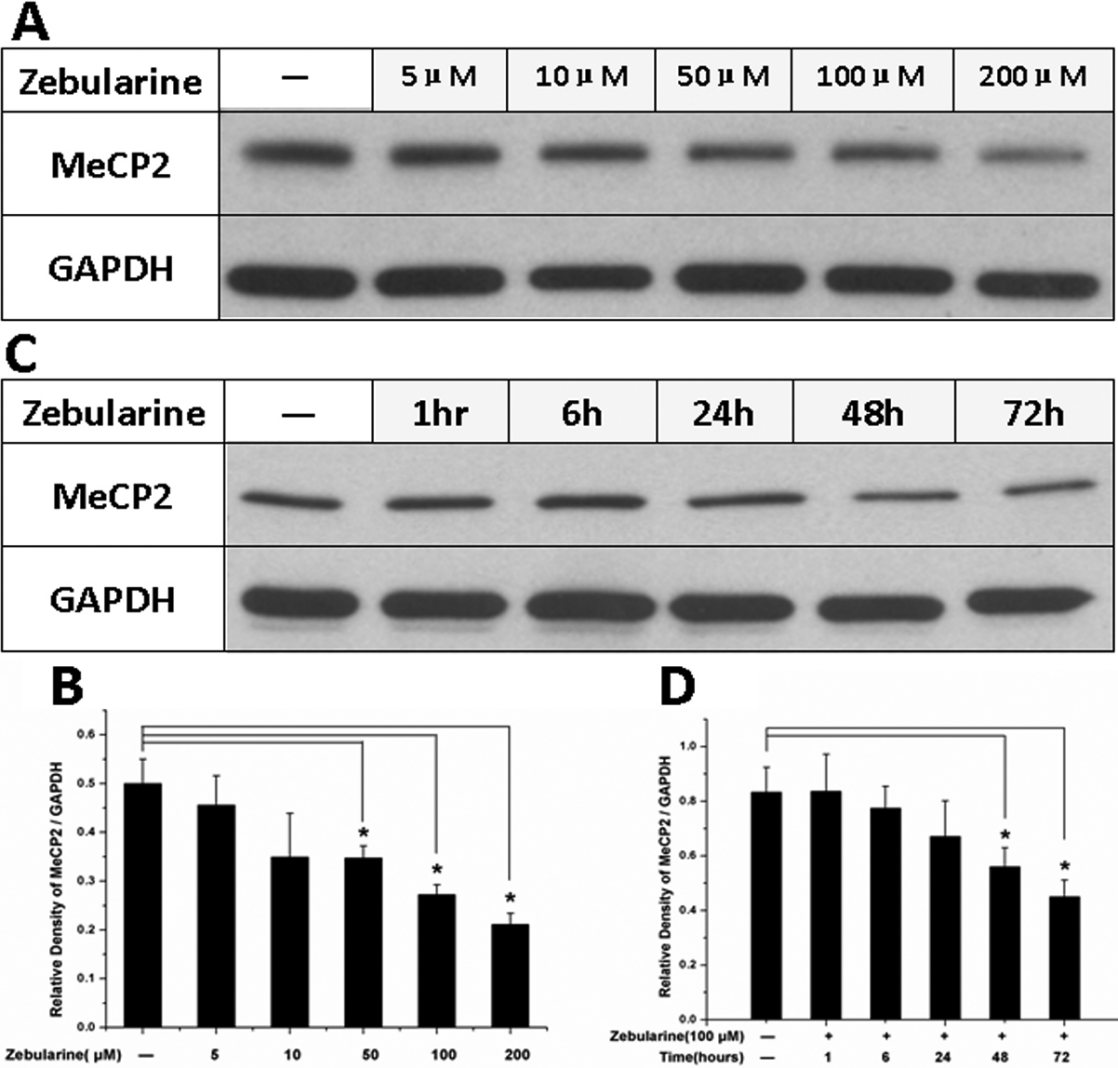
Zebularine decreased MeCP2 expression in LECs. **A**: Zebularine inhibited MeCP2 expression in a dose-dependent pattern. As the concentration of zebularine increased from 5 μM to 200 μM, the MeCP2 protein level decreased to 40% of the control level after zebularine treatment for 72 h. **C**: Zebularine also inhibited MeCP2 expression in a time-dependent pattern. After 1 to 72 h of treatment with 100 μM zebularine, the MeCP2 protein level gradually decreased to 55% of the control level. Densitometry analysis of three independent western blots shows quantitation of MeCP2 levels (**B**, **D**). *p<0.05. The error bar indicates mean±SEM.

In this study, we attempted to find a reagent to inhibit both TGFβ-induced MeCP2 and α-SMA expression. We have found that zebularine could inhibit the expression of MeCP2 in retinal pigment epithelial cells (data not published). In this study, we analyzed zebularine treatment on MeCP2 expression in lens epithelial cells. Our results indicate that zebularine inhibits MeCP2 expression in a dose-dependent pattern. As the concentration of zebularine increased from 5 μM to 200 μM, the MeCP2 protein level decreased to 40% of untreated controls after zebularine treatment for 72 h (Figure 3A,B). Zebularine also inhibited MeCP2 expression in a time-dependent pattern. After 1 to 72 h of treatment with 100 μM zebularine, the MeCP2 protein level gradually decreased to 55% of the control level (Figure 3C,D).

### Zebularine suppressed TGFβ2-induced α-SMA expression

**Figure 4:**
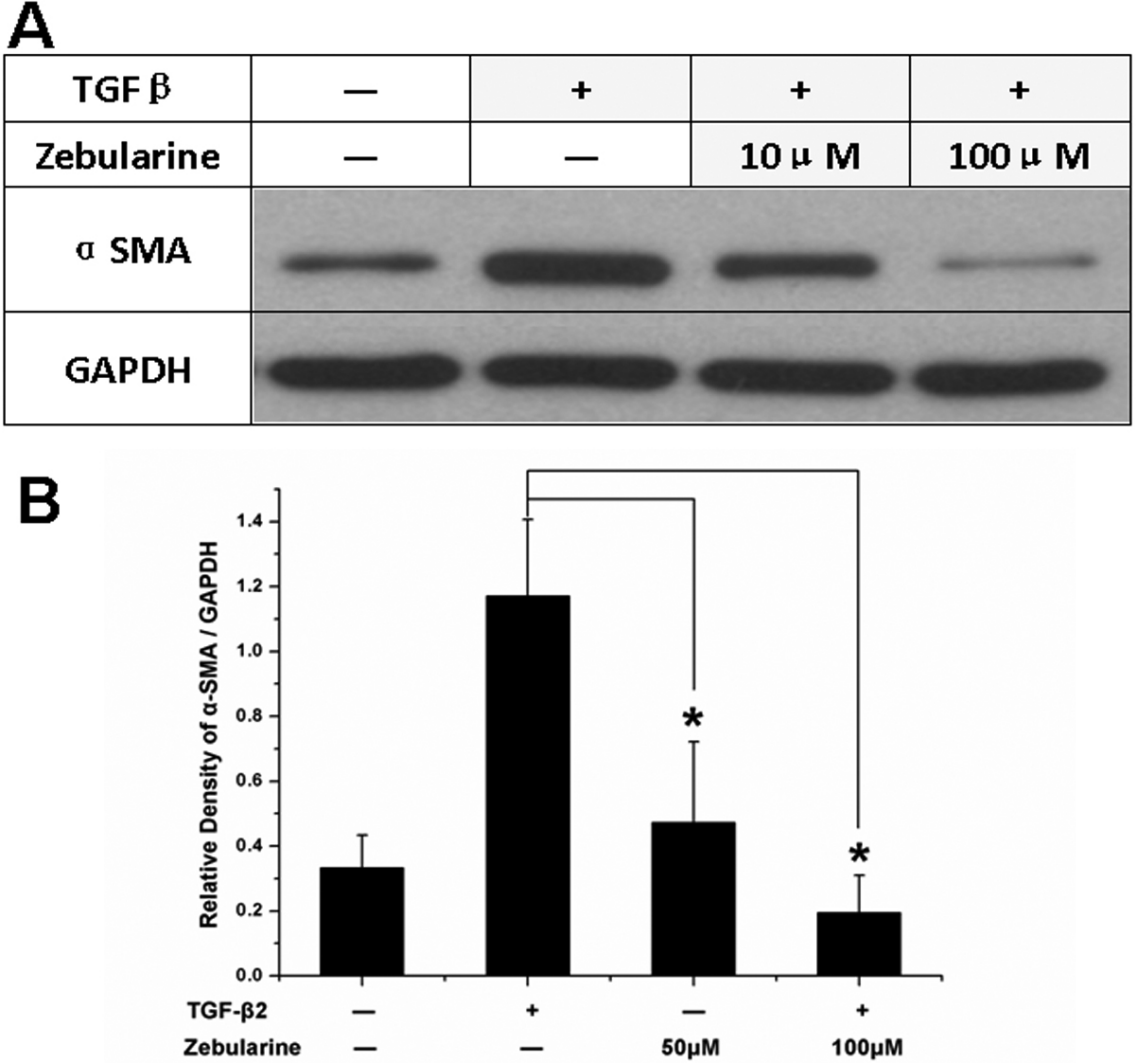
Zebularine suppressed TGFβ2-induced α-SMA expression. **A**: After TGFβ2 treatment, the α-SMA protein level increased by 3.5 fold in 72 h. However, the α-SMA remained at a low level if treated with zebularine and TGFβ2 together. **B**: Densitometry analysis of three independent western blots shows the quantitation of α-SMA levels. *p<0.05. The error bar indicates mean±SEM.

Moreover, we investigated whether zebularine also had an effect on TGFβ2-induced α-SMA expression. After treatment with TGFβ2, the α-SMA protein level increased 3.5 fold in 72 h. However, the α-SMA remained at a low level if it was treated with zebularine and TGFβ2 together (Figure 4).

## Discussion

In this study, we found that MeCP2 and α-SMA were co-expressed in human PCO membranes. MeCP2 played an important role in TGFβ2-induced α-SMA expression in lens epithelial cells. Zebularine could inhibit MeCP2 expression and could also suppress the TGFβ2-induced α-SMA expression.

DNA methylation is a key epigenetic regulatory mechanism that plays an important role in *α-SMA* gene expression [10]. Recently, Hu et al. [11] reported that MeCP2 binds to *α-SMA* and plays a direct activator role for the *α-SMA* gene in lung fibroblasts. In the current study, we found that TGFβ2 could not induce *α-SMA* expression without MeCP2, which is consistent with the Hu et al. [11] report. Therefore, MeCP2 played an important activator role in TGFβ2-induced LEC epithelial-mesenchymal transition.

The potential mechanisms by which MeCP2 regulates *α-SMA* activation are complex. One possibility is that MeCP2 binds to the *α-SMA* promoter and directly activates its expression. Initially, MeCP2 was known as a transcriptional repressor that functions by binding to methylated CpG islands present in the target gene sequence [20]. However, subsequent studies reveal that only 6% of the MeCP2 binding sites are in CpG islands. Most of MeCP2-bound promoters belong to actively expressed genes [21]. Thus, although MeCP2 preferentially binds to methylated DNA, it has the potential to act as both a repressor and an activator of gene expression [11,12]. Alternatively, MeCP2 may regulate *α-SMA* through peroxisome proliferator-activated receptor gamma (PPARγ), which is a repressor of *α-SMA* expression. PPARγ is a nuclear receptor protein that regulates the expression of genes and plays essential roles in the regulation of cellular differentiation. MeCP2 represses *PPARγ* transcription by suppressing both transcriptional initiation and elongation [22]. Therefore, in response to TGFβ, MeCP2 may activate the expression of *α-SMA* by inhibiting *PPARγ*.

The essential role that MeCP2 plays in TGFβ2-induced *α-SMA* expression provides a potential target for inhibiting the epithelial-mesenchymal transition in LECs. Zebularine has been reported to inhibit *MeCP2* expression in some cell lines [23]. In preliminary studies, we found that zebularine suppresses the expression of *MeCP2* in retinal pigment epithelial cells (data not published). Therefore, zebularine was selected for further analysis.

Recent phase I clinical studies showed that the methylation inhibitor is well tolerated and safe [24-26]. Further phase II clinical trials are in progress. The methylation inhibitor is a new strategy for treating disease. The focus of our study, zebularine, is a DNA methylation inhibitor. Other DNA methylation inhibitors, such as 5-Aza-C and 5-Aza-dC, have shown significant clinical activity against tumors. However, the clinical use of these agents is complicated by their toxicity and instability in aqueous solutions [17]. The half life of zebularine is much longer, and its toxicity is much lower. Therefore, zebularine offers a better option for potential clinical application.

**Figure 5:**
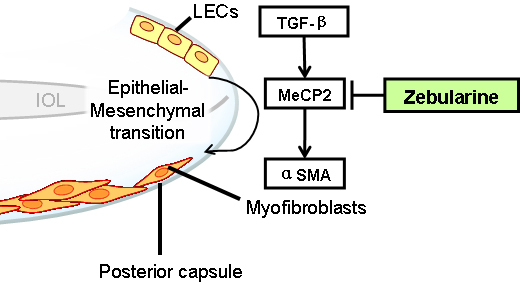
Model for zebularine inhibition of TGFβ2-induced LEC-myofibroblast transdifferentiation. MeCP2 plays an important role in TGFβ-induced, LEC-myofibroblast transdifferentiation, which is an event marked by α-SMA expression. Zebularine inhibited not only MeCP2 but also TGFβ-induced α-SMA expression. Therefore, zebularine could potentially prevent PCO formation. LEC=lens epithelial cells; IOL=intra ocular lens.

In this study, we found that zebularine suppresses the expression of *MeCP2* in a time- and dose-dependent pattern in LECs. Furthermore, zebularine suppressed TGFβ2-induced *α-SMA* expression (Figure 5). Therefore, zebularine is a promising treatment for the possible prevention of PCO after conventional cataract surgery.
